# Opioid-Free Perioperative Analgesia for Revision Total Hip Arthroplasty in a Patient With a History of Substance Use Disorder

**DOI:** 10.1155/cria/5521332

**Published:** 2025-03-06

**Authors:** Robert L. Owen, Mitchell J. Kerfeld, Jason K. Panchamia, David A. Olsen, Adam W. Amundson

**Affiliations:** Department of Anesthesiology and Perioperative Medicine, Mayo Clinic, Rochester, Minnesota 55905, USA

**Keywords:** opioid free, opioid sparing, opioid use disorder, PENG block, peripheral nerve block, regional anesthesia, revision total hip arthroplasty, SIFI block

## Abstract

**Background:** Given the endemic opioid crisis in our communities, there is an important need for opioid-sparing analgesia alternatives for invasive surgical procedures that use multimodal analgesia to help reduce or avoid opioid consumption perioperatively. Unfortunately, a significant challenge exists in reducing opioid consumption with procedures that are known to cause a significant amount of postoperative discomfort. Regional anesthesia via peripheral nerve blockade is a modality that can take advantage of pertinent anatomy, greatly reduce a patient's postoperative pain, and minimize opioid consumption. Some anatomical locations, such as the hip joint, have complex innervation. For such anatomical locations, a single peripheral nerve block may fail to cover all the involved sensory nerves, and thus, incorporating additional regional blocks can synergistically provide sufficient analgesic coverage.

**Case Presentation:** A 69-year-old man presented for revision total hip arthroplasty. Due to his previous history of opioid abuse, he requested an opioid-free perioperative experience. The patient's past medical history was complex, and he had suffered recurrent prosthetic joint infections. To accomplish opioid-free perioperative analgesia for his revision hip arthroplasty, we utilized both the suprainguinal fascia iliaca block and the pericapsular nerve group block, in combination with surgeon-administered local infiltration analgesia and oral and intravenous nonopioid systemic pain medications. The patient was successfully able to avoid opioids through the procedure and postoperative course, with the majority of his pain scores being zero out of 10 during his hospital admission.

**Conclusions:** Complimentary regional nerve blocks, when combined with surgeon-administered local infiltration analgesia and multiple systemic nonopioid medications, can provide sufficient analgesia to cover the complexly innervated hip joint and accomplish an opioid-sparing revision total hip arthroplasty perioperative course.

## 1. Introduction

Opioid-sparing techniques have been associated with excellent analgesia, early mobilization, and faster hospital discharge times in orthopedic surgery. For primary total hip arthroplasty (THA), the utilization of the suprainguinal fascia iliaca (SIFI) block, the pericapsular nerve group (PENG) block, and surgeon-administered local infiltration analgesia (LIA; also called periarticular injection) all have individually been shown to promote early physical therapy and improve patient outcomes [1–5]. However, pain management following a revision THA can be unpredictable and difficult to manage due to the varying complexities of each surgical case and unique patient characteristics. Although regional anesthetic techniques, such as the SIFI and PENG blocks, have individually been shown to reduce opioid consumption following THA [3–5], the possibility of combining regional anesthetic techniques to appropriately cover the cutaneous and bony innervation of the entire hip joint while averting perioperative opioid use has not been described. Furthermore, the sparse studies that do exist regarding opioid-sparing approaches to THA only demonstrate the avoidance of opioids during the intraoperative phase of care but neglect to demonstrate full avoidance of opioids during the postoperative phase [6, 7]. We present a challenging case of a patient with a history of opioid addiction who underwent a complex revision THA for recurrent prosthetic joint infections. The patient's request for an opioid-free perioperative experience required a synergistic combination of regional analgesic techniques, as well as a multidisciplinary team approach between our anesthesia team and our surgical colleagues, to be able to meet the patient's expectations.

## 2. Case Presentation

A 69-year-old man with a complex medical history presented to a large academic hospital for revision THA after unsuccessful treatment of recurrent periprosthetic joint infections. The patient was American Society of Anesthesiologists physical status III with comorbidities including diabetes mellitus type 2, moderate aortic valve stenosis, chronic kidney disease stage 3, and class III obesity. An evaluation by the presurgical clinic revealed a history of opioid use disorder and a strong desire by the patient to maintain sobriety. Given the patient's concern for relapse and motivation to avoid opioids, the anesthesia team proposed a comprehensive multimodal pain regimen entailing nonopioid analgesic medications plus the combination of the SIFI and PENG nerve block techniques with LIA to provide overlap and synergy in analgesic coverage. Of note, due to the patient's history of recurrent prosthetic infections and concern for seeding infection, we elected not to perform a lumbar plexus (psoas) block or use an indwelling catheter.

Preoperatively, the patient received 1000 mg of acetaminophen and 400 mg of celecoxib orally. Nerve block procedural sedation consisted of intravenous midazolam 2 mg. In the supine position, a wide prep and drape accommodated both the SIFI and PENG blocks. The SIFI was performed using a Fujifilm SonoSite X-Porte HFL38 linear ultrasound transducer. A Braun 20-gauge 100 mm insulated needle was advanced in-plane to enter the fascia iliaca cranial to the pelvic brim, followed by fascial plane hydrodissection. Dynamic ultrasound imaging confirmed local anesthetic spread under and throughout the fascia iliaca plane with the deep circumflex artery in view superficially [1]. A total of 35 mL of 0.25% bupivacaine with 1:200,000 epinephrine was injected.

Next, the PENG block was performed using the same equipment with the ultrasound transducer aligned with the pubic ramus. The needle was then advanced in-plane from laterally to medially, with the needle tip positioned in the musculofascial plane between the psoas tendon and the pubic ramus [2]. After negative aspiration for blood was confirmed, 15 mL of 0.25% bupivacaine with 1:200,000 epinephrine was injected in divided doses.

**Figure 1:**
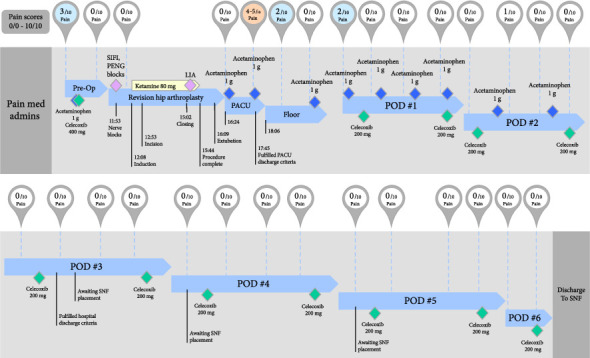
Perioperative timeline with pain scoring in relationship to nonopioid analgesia administrations. Pre-Op = preoperative period, SIFI = suprainguinal fascia iliaca block, PENG = pericapsular nerve group block, LIA = surgeon-administered local infiltration analgesia, PACU = postanesthesia care unit, POD = postoperative day, SNF = skilled nursing facility.

For the procedure, the patient underwent general anesthesia with an endotracheal tube and received a total of 80 mg of intravenous ketamine for intraoperative analgesia. General anesthesia was selected over spinal anesthesia due to the anticipated longer duration of surgery, high likelihood of large intraoperative blood loss, and infection risk. The surgeon used an anterior extended trochanteric osteotomy approach. The case lasted 171 min (2 h and 51 min), with estimated blood loss reported as 2000 mL. During wound closure, the surgeon performed LIA by injecting 60 mL consisting of 150 mg of ropivacaine, 15 mg of ketorolac, and 100 μg of epinephrine methodically around the joint and skin [8]. In the postanesthesia care unit, the patient's numeric rating scale pain score was zero (Figure 1) with appropriate sensory and motor blockade, and he received no additional pain medications. He fulfilled discharge criteria from the postanesthesia care unit after 81 min and was subsequently transferred to the orthopedic inpatient floor.

On postoperative day (POD) #1, he continued to have a pain score of 0/10 in the surgical extremity for the majority of the day (Figure 1). He regained full motor function the morning of POD #1 and began receiving physical therapy. His sensory blockade lasted until the evening of POD #1. The postoperative pain regimen consisted of scheduled acetaminophen 1000 mg every six hours (which was no longer needed after POD #2) and celecoxib 200 mg twice daily. He successfully remained opioid-free throughout his entire perioperative hospitalization with rare, short-lived mild deviations from 0/10 pain during the first two postoperative days that were fully responsive to the scheduled medications. The patient fulfilled discharge criteria on POD #3 with 0/10 pain that only needed celecoxib 200 mg twice daily. Of note, the patient was not discharged until POD #6 (Monday) due to the timing of rehabilitation facility placement after the weekend. During this time, all his pain scores on POD #3–#6 were 0/10. He was discharged to a skilled nursing facility for physical therapy and rehabilitation on POD #6 with 0/10 pain at the time of dismissal. His discharge pain medication regimen consisted of continued scheduled celecoxib 200 mg by mouth twice daily for an additional 8 days to complete 2 weeks, as well as having available acetaminophen 1000 mg four times daily as needed.

## 3. Discussion

The patient's request was to undergo a complex surgical revision of his infected hip while remaining opioid-free during his perioperative recovery. This was accomplished through a multimodal pain management strategy consisting of complementary regional anesthesia techniques, which allowed for analgesic overlap to cover the hip joint synergistically and comprehensively, surgeon-administered LIA, and various oral and intravenous nonopioid analgesic systemic medications.

Due to the complex innervation of the hip joint, sufficient analgesic coverage cannot be obtained by a single peripheral or fascial plane nerve block. Bony and cutaneous sensory innervation of the hip originates from the femoral, obturator, lateral femoral cutaneous, superior gluteal, and sciatic nerves. The anterior hip capsule is mainly innervated by the femoral and obturator nerves, with occasional contribution from the accessory obturator nerve. Articular branches of the femoral nerve provide most of the innervation to the lateral and superomedial capsule, while the articular branches of the obturator supply the inferomedial aspect [3, 9]. Surgeon-administered LIA is a proven postoperative pain modality; however, its drawback includes inconsistent neural blockade of the articular branches that contribute to pain after hip replacement [8].

While the literature does show that the SIFI and PENG blocks reduce opioid consumption independently in THA [1–5], there is sparse evidence demonstrating the analgesic efficacy of combining peripheral nerve blocks to sufficiently cover the hip joint to avoid opioid use during revision THA. We surmise that the complementary nature of each of these two blocks serves to help cover the areas that the other is lacking. The SIFI block is an anterior approach to the lumbar plexus in the space between the iliacus muscle and the fascia iliaca. It targets the femoral, obturator, and lateral femoral cutaneous nerves, which lie in this space [4, 10]. We favored the SIFI block over other fascia iliaca compartment blocks that are performed at or below the inguinal ligament since the SIFI block's suprainguinal position has been shown to decrease opioid consumption after THA by providing more consistent efficacy in anesthetizing the lateral femoral cutaneous nerve as well as anterior cutaneous femoral nerve branches [1, 5]. Unfortunately, the SIFI block has limitations, one of which includes sparing of the obturator nerve [1]. As such, the PENG block complimentarily targets articular branches of the accessory obturator that the SIFI block misses, as well as articular branches of the femoral nerve in the anterior hip joint. Additionally, the PENG block spares some cutaneous structures that the SIFI block covers. Although the PENG block can provide effective analgesia following hip fractures [2], it can also supplement other regional anesthesia techniques for improved analgesic coverage following THA [3].

Ubiquitous opioid abuse has been well-documented in the United States [11, 12], with particular concern identified for those recovering from surgery [13–15] and for those who are opioid-naïve [16]. Furthermore, opioid abuse has been shown in orthopedic patients to a much greater degree than the national prevalence of opioid abuse [13]. There is a significantly increased risk of postoperative morbidity and mortality with preoperative opioid use and perioperative opioid misuse, as well as increased healthcare costs [13]. These and other endemic realities underscore the need for the continued pursuit of opioid alternatives to surgical pain management.

In conclusion, understanding the anatomical innervation of the hip joint is critical for a targeted peripheral nerve block approach for an opioid-sparing perioperative multimodal analgesia for complex surgical procedures. The complementary combination of the SIFI and PENG blocks, along with LIA, synergistically targeted most of the sensory innervation of the hip joint and, when combined with a multimodal approach, allowed this patient to receive an opioid-free revision THA perioperative course.

## Data Availability

Data sharing is not applicable to this article as no datasets were generated or analyzed during the current study.

## Nomenclature

THA: Total hip arthroplasty
SIFI: Suprainguinal fascia iliaca block
PENG: Pericapsular nerve group block
LIA: Surgeon-administered local infiltration analgesia
POD: Postoperative day
